# Childhood psychological abuse and relational aggression among adolescents: A moderated chain mediation model

**DOI:** 10.3389/fpsyg.2022.1082516

**Published:** 2023-01-18

**Authors:** Ting Li, Yuhuai Huang, Meiru Jiang, Shutao Ma, Yankun Ma

**Affiliations:** ^1^School of Education, Guangzhou University, Guangzhou, China; ^2^Nansha Tianyuan Campus of Guangzhou High School, Guangzhou, China; ^3^Qionglai West Street Primary School, Sichuan, China

**Keywords:** childhood psychological abuse, relational aggression, rejection sensitivity, relational victimization, cognitive reappraisal

## Abstract

**Introduction:**

Adolescents are in a period when a marked upward trend of adolescents relational aggression. Even though previous studies have found that childhood psychological abuse experience is an important factor influencing adolescent relational aggression, it is unclear when and under what circumstances childhood psychological abuse impacts adolescent relational aggression. This study constructed a moderated chain mediation model to investigate the influence of childhood psychological abuse on relational aggression among Chinese adolescents and its internal mechanism.

**Methods:**

Data from 1868 (923 male and 945 female, M = 14.31, SD = 1.60) Chinese adolescents in two full-time middle schools in Guangzhou were collected via a cross-sectional survey in 2020. Adolescents reported on childhood psychological abuse, relational aggression, rejection sensitivity relational victimization and cognitive reappraisal.

**Results:**

The results demonstrated that: (1) childhood psychological abuse was significantly positively related to relational aggression; (2) childhood psychological abuse was significantly linked with adolescents’ relational aggression through the separate mediating effects of rejection sensitivity and relational victimization, as well as through the chain mediating effects of rejection sensitivity and relational victimization; (3) the chain mediated effect of childhood psychological abuse on relational aggression through rejection sensitivity and relational victimization was moderated by cognitive reappraisal.

**Conclusion:**

These findings indicate that childhood psychological abuse, as a kind of poor parenting style, has influence on adolescents’ internal personality (rejection sensitivity) and external behavior development (relational victimization and relational aggression). This study is helpful to demonstrate the protective effect of cognitive reappraisal and reveal the internal mechanism of childhood psychological abuse on relational aggression.

## Highlights

- Childhood psychological abuse was significant positively related to adolescents’ relational aggression.
- Childhood psychological abuse was significantly linked with adolescents’ relational aggression through the separate mediating effects of rejection sensitivity and relational victimization, as well as through the chain mediating effects of rejection sensitivity and relational victimization.
- The chain mediated effect of childhood psychological abuse on relational aggression through rejection sensitivity and relational victimization was moderated by cognitive reappraisal.

## Introduction

Adolescents are in a critical period of rapid physical and mental development, there is a marked upward trend in the importance of maintaining fine peer relationships and social status (Liu et al., 2011). Relational aggression refers to a series of behaviors that the attacker deliberately destroys the peer relationship of the attacked by means of exclusion, rumor, and social manipulation (Crick and Grotpeter, 1995). In comparison to direct aggression, relational aggression is a form of campus violence that is intangible and characterized by concealment. Individuals who suffer from relational aggression will not only further deteriorate their own interpersonal relationships, but also may have direct aggression and other behaviors, and their physical and mental health development will suffer severe and long-lasting detrimental effects (Lei et al., 2019; Wang and Yu, 2019). In light of this, there is an urgent need to comprehend the factors that influence relational aggression among adolescents.

### Childhood psychological abuse and relational aggression

General aggression model is the most extensive model to explain the mechanism of relational aggression. According to the model, the generation of individual aggression behavior can be explained by environmental factors (such as family) and individual factors (such as personality) (Kowalski et al., 2014). Among environmental factors, childhood psychological abuse, as the family environment experienced by adolescents at the earliest stage, has a direct and long-term impact on their subsequent cognitive and behavioral development. Adverse childhood experiences, including many different kinds of child maltreatment (such as physical or psychological neglect and abuse), and adverse parenting styles are risk predictors of both internalized (Barber et al., 2014; Balistreri and Alvira-Hammond, 2016) and externalized problems (Perez et al., 2018; Mumford et al., 2019) in adolescents.

Previous studies have found that childhood psychological abuse experience is an important factor influencing adolescent relational aggression (Crawford and Wright, 2007). 36% of the world’s children are affected by childhood psychological abuse, which is defined as the long-term use of intimidation, denigration, meddling, connivance, and neglect by the primary caregivers of children in inappropriate ways to raise children (Stoltenborgh et al., 2012). This parenting style can have a substantial impact on adolescents’ behavioral disorders (Afifi et al., 2011) and aggressive behavior (Harford et al., 2014). Individual emotion or behavior can be transferred from one environment to another, according to the spillover hypothesis (Almeida et al., 1999), as evidenced by the acquisition of certain aggressive behavior, authoritarian behavior, or psychological control by children from their parents, and apply these behaviors to situations outside the home, where they become aggressors themselves. Studies have demonstrated that negative parenting styles (such as authoritarian, permissive) can significantly predict relational aggression in children (Casas et al., 2006). Experience of psychological abuse is a strong predictor of individuals’ problem behaviors (Liu et al., 2019) and aggressive behaviors (Jin and Wang, 2017; Sun et al., 2017a, b). Therefore, this study proposed the hypothesis:

*Hypothesis 1* (*H1*): Childhood psychological abuse is significantly positive related to relational aggression.

It is unclear, however, when and under what circumstances childhood psychological abuse impacts adolescent relational aggression. Therefore, we need to further explore the internal mechanism of action between childhood psychological abuse and relationship aggression. Considering that rejection sensitivity and relational victimization were correlated with childhood psychological abuse (Erozkan, 2015) and relational aggression (Zimmer-Gembeck and Duffy, 2014), and cognitive reappraisal can significantly alleviate the frustration emotional response caused by rejection sensitivity (Zuo et al., 2016). This study intends to take rejection sensitivity and relational victimization as mediating variables and cognitive reappraisal as moderating variables to further discuss the influence of childhood psychological abuse and relational aggression.

### The chain mediating effect of rejection sensitivity and relational victimization

Rejection sensitivity is a negative personality tendency, which refers to the anxious anticipation, the perception of accommodation and the intense emotional behavior reaction of individuals to possible rejection in interpersonal communication (Downey and Feldman, 1996). Studies have found that childhood psychological abuse may be one of the most significant variables influencing rejection sensitivity (Erozkan, 2015). On the one hand, attachment theory asserts that if children are often rejected and neglected by their main caregivers in their early life, their psychological needs cannot be met and they are prone to develop an insecure attachment pattern, which will make them more sensitive to rejection cues in interpersonal communication. Empirical studies support the theory that parental violence significantly and positively predicts rejection sensitivity in children (Brendgen et al., 2002). Erozkan (2009) also found that poor parental style leads to individuals being in a situation of long-term stress and rejection, thus developing high rejection sensitivity. On the other hand, according to the theory of frustration - attack, attack behavior is caused by the setbacks in the individual, the individual perceived from others’ refusal (Berkowitz, 1989) is a common human setbacks, refused to high sensitivity of teenagers are easily in interpersonal communication signal for hostile to explain, so feel more personal setbacks, triggering more attacks (Bondü and Krahé, 2015). From the defensive motivational system, in the case of possible rejection, individuals with high rejection sensitivity are more likely to activate the self-protection mechanism and exhibit aggression (Downey et al., 2004; Romero-Canyas et al., 2010). Research supports the aforementioned theoretical perspectives. In a longitudinal study, Zimmer-Gembeck et al. (2016) found that adolescents with high rejection sensitivity are more likely to have angry emotions and participate in retaliatory conduct when facing rejection information, that is, to carry out more explicit and relational aggression behaviors. Therefore, this study hypothesized that:

*Hypothesizes 2a* (*H2a*): Rejection sensitivity mediates the relationship between childhood psychological abuse and relational aggression.

Meanwhile, relational victimization, as a subtype of peer victimization, can significantly predict adolescents’ relational aggression (Ji et al., 2012). Relational victimization is when individuals endure relational attacks from peers in interpersonal communication, resulting in specific damage to their social standing and friendships (Crick et al., 2002). On the one hand, according to attachment theory (Bowlby, 1969), abusive experiences inhibit the development of the secure attachment model in individuals, and individuals apply the negative emotional reactions and hostility engendered by abusive experiences to peer situations, resulting in increased peer victimization of individuals (Zhu et al., 2019). Past studies have shown that individuals who have been abused in the past lack social skills and may therefore have difficulty dealing with peer conflict and experience more ostracism and aggression (Ohene et al., 2006). Shields and Cicchetti (2001) also found that childhood psychological abuse was a risk factor for peer victimization. Interpersonal risk model (Kochel et al., 2012) denotes that for adolescents, a lack of solid interpersonal relationships or being attacked by others are significant stress events that cannot be disregarded, leading to a succession of adverse consequences such as problem behaviors (Light et al., 2013). A meta-analysis showed a strong association between relational victimization and relational aggression (Casper et al., 2020). On this basis, this study hypothesized that:

*Hypothesizes 2b* (*H2b*): Relational victimization mediates the relationship between childhood psychological abuse and relational aggression.

On the other hand, based on the developmental systems theory, both individual internal factors and peer factors may play a role in the relationship between family factors and individual behavior. That is to say, adolescents who experience childhood psychological abuse in adverse family environments will first affect their own cognition, making individuals more sensitive to rejection cues in interpersonal communication, and thus more likely to suffer peer victimization in interpersonal communication (Jacobs and Harper, 2013). Multiple studies have demonstrated that relational victimization is a significant predictor of future violations and aggressive behavior (Sullivan et al., 2006). Based on the foregoing theoretical and empirical research, this study hypothesized that:

*Hypothesizes 2c* (*H2c*): Rejection sensitivity and relational victimization play a chain mediating role between childhood psychological abuse and relational aggression.

### The moderating effect of cognitive reappraisal

In addition, studies have found that cognitive reappraisal, as a positive emotion regulation strategy, can mitigate the impact of negative life events on individuals (Zuo et al., 2016; Wang et al., 2020). Previous empirical studies also have shown that cognitive reappraisal can effectively reduce individuals’ aggressive behaviors by replacing automated hostile explanations (rejection sensitivity) with reasonable and non-hostile explanations (Denson, 2015; Hou et al., 2017). Cognitive reappraisal refers to that when individuals are faced with negative events that may trigger emotions, they alter their cognition of the event and diminish its negative meaning to the individual in order to avoid the generation of negative emotions (Gross and John, 2003). According to the emotional regulation process model, individuals who are good at using cognitive reappraisal can face negative emotional events with a more positive attitude, so as to weaken the adverse influence of emotional events on their inner psychological quality. An empirical study demonstrated that cognitive reappraisal moderated the mediating effect of shame between social exclusion and adolescent self-injury, and that the detrimental impact of social exclusion on individual shame was mitigated when the individual’s cognitive reappraisal level was high (Wang et al., 2020). Considering that cognitive reappraisal, as an antecedent focus strategy, generally occurs at the early stage of emotion generation (Buhle et al., 2014), and has a significant easing effect on frustration emotional responses generated by rejection sensitivity (Zuo et al., 2016). This study hypothesizes that (H3):

*Hypothesizes 3* (*H3*): Cognitive reappraisal can moderate the first half of the chain mediation path (childhood psychological abuse → rejection sensitivity), Specifically, adolescents with a higher level of cognitive reappraisal can mitigate the negative effects of childhood psychological abuse to rejection sensitivity.

### Current study

**Figure 1:**
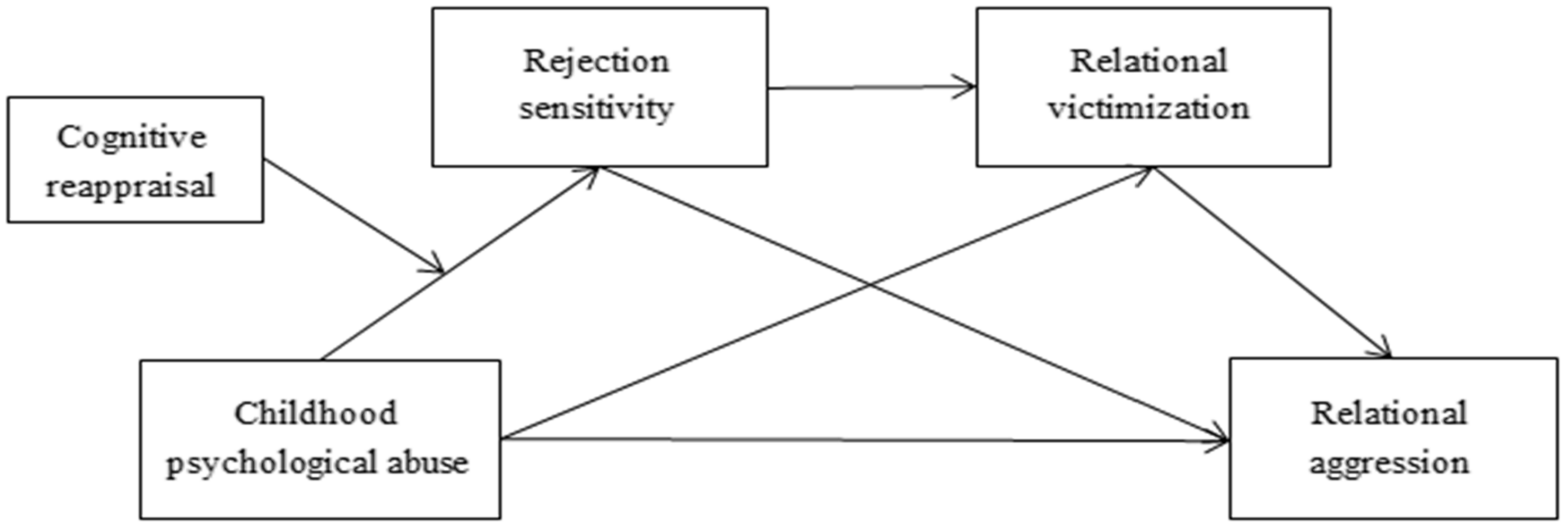
The hypothetical theoretical model.

Based on the aforementioned theories and research, the purpose of this study is to investigate the chain mediating effect of rejection sensitivity and relational victimization on the association between childhood psychological abuse and adolescent relational aggression, as well as the moderating effect of cognitive reappraisal. The hypothetical theoretical model is exhibited in Figure 1.

## Method

### Participants

This study investigated 1,966 middle school students from two full-time middle schools in Guangzhou employing cluster sampling. With the approval of the students and their parents, the test was administered to the class as a unit, and 1,868 valid questionnaires with a recovery rate of around 95% were gathered. In the effective sample, there were 923 boys and 945 girls, aged from 12 to 17 years old (*M* = 14.31, *SD* = 1.60).

## Measures

### Childhood psychological abuse

In the form of a recall scale, the Child Psychological Maltreatment Scale (Pan et al., 2010) was used to measure the level of childhood psychological abuse among middle school students. There are 23 items in this scale, and participants were asked to respond based on their childhood experiences (such as “my parents scolded me for no reason”). The participants were asked to respond to items on a 5-point Likert-type scale, ranging from 0 = never to 4 = all the time. The Cronbach’s α coefficient of the scale in this study is 0.92.

### Relational aggression

The Chinese revision of the Self-rated Relational Aggression Questionnaire (Liang, 2005) established by Werner and Crick (1999) and adapted by Loudin et al. (2003) was used to measure relational aggression among middle school students. The questionnaire contained nine items, and participants were asked to respond based on their experiences over the 6 months (such as “when someone annoys me, ignore him/her for a short time”). The participants were asked to respond using a 5-point Likert-type scale, ranging from 0 = never to 4 = all the time. After all items were scored in reverse, the relational attack level increased proportionally to the subject’s score on all items. The Cronbach’s α coefficient of the questionnaire in this study is 0.88.

### Rejection sensitivity

The Children’s Rejection Sensitivity Questionnaire (Downey et al., 1998) was utilized to measure the levels of rejection anxiety, rejection anger and rejection expectation of middle school students. Each of the 12 peers and teacher scenarios contained three questions. The questionnaire was scored by 6 points, and the score calculation method was as follows: rejection anxiety dimension = rejection anxiety × rejection expectation; Anger rejection dimension = anger rejection × expectation rejection. Combining the rejection anxiety dimension and the anger rejection dimension yields the overall rejection sensitivity score, and the higher the score, the greater the rejection sensitivity. The Cronbach’s α coefficient of the questionnaire in this study is 0.86.

### Relational victimization

Children’s exposure to peer victimization was measured using the Relational Victimization Subscale (Zhang et al., 2009) in the Multidimensional Peer Victimization Scale (MPVS) developed by Mynard and Joseph (2000). There were 8 items in the scale, and participants were asked to respond based on their experiences over the past 6 months (such as “nearly half a year, other students deliberately do something to let the teacher do not like me”). The scale was scored by 4 points, and the higher the score, the greater the degree of relationship infringement. The Cronbach’s α coefficient of the scale in this study is 0.90.

### Cognitive reappraisal

The Cognitive Reappraisal Strategy Subscale (Wang et al., 2007) of the Emotional Regulation Strategy scale compiled by Gross and John (2003) was used to assess the level of cognitive reappraisal strategies employed by middle school students. The scale contained seven items, and participants were asked to respond based on their experiences over the 6 months (such as “I change the way I interpret situations to control my emotions”). The participants were asked to respond using a 5-point Likert-type scale, ranging from 1 = never to 5 = all the time. The frequency with which respondents employed the cognitive reappraisal strategy was proportional to their score. The Cronbach’s α coefficient of the scale in this study was 0.89.

### Procedure

Before conducting the survey, the consent of teachers, participants, and their guardians was secured. One or two graduate students majoring in psychology were responsible for the distribution and collection of questionnaires. All respondents were informed that their participation was entirely voluntary and that the results would be utilized exclusively for scientific research. In addition, our testing material and survey procedures were approved by the Ethics in Human Research Committee of School of Education, Guangzhou University.

In this study, SPSS 22.0 (IBM, Armonk, NY, USA) was used to input and process the data, and the macro program PROCESS v4.0 (^1^ accessed on 25 May 2022) plug-in compiled by Hayes (Hayes, 2017) and Mplus 7.4 was utilized to examine a moderated chain mediation model. The analyses were performed in the following four aspects. First, the independent sample *T*-test, descriptive statistics and correlation analysis were conducted for each variable. Second, using 2,000 bootstrap samples and the PROCESS macro (Model 6), we tested the chain-mediating effect of rejection sensitivity and relational victimization in the link between childhood psychological abuse and relational aggression. The parameter was statistically significant if the confidence interval excluded 0. Third, according to the suggestions of Wen and Ye (2014), we used Mplus7.4 to examine the moderating effect of cognitive reappraisal on the chain mediation models with 2,000 bootstrap samples. All variables were standardized prior to the formal data processing. Finally, we used Mplus7.4 to analyses alternate model for greater confidence in the proposed direction of associations between the childhood psychological abuse and relational aggression. For all model, good model fit was determined by the following criteria: comparative fit index (CFI) > 0.90; Tucker-Lewis index (TLI) > 0.90; standardized root mean residual (SRMR) < 0.08; and root mean square error of approximation (RMSEA) <0.05.

## Results

### Common method bias

The data acquired for this study came from self-reports. Although for some items reverse scoring was utilized to control the bias generated by the effect of the common method, additional bias testing of the common method was still required. Therefore, exploratory factor analysis was undertaken on the variables using the Harman single factor method test (Zhou and Long, 2004). The results revealed 11 common factors with characteristic root values greater than 1, and the highest variation explained by common factors was 19.26 percent, which was less than the critical value of 40 percent. Therefore, there was no significant common method bias in this study.

### Descriptive statistics

The independent sample T-test results of the gender and age of the subjects among the model variables were shown in Table 1. Of all the variables, the level of childhood psychological abuse [*t*_(1866)_ = 4.238, *p* < 0.001], relational victimization [*t*_(1866)_ = 6.013, *p* < 0.001] and cognitive reappraisal [*t*_(1866)_ = −5.323, *p* < 0.001] proved to be significantly different among age; the level of childhood psychological abuse [*t*_(1866)_ = −3.324, *p* < 0.01] and rejection sensitivity [*t*_(1866)_ = −7.732, *p* < 0.001] proved to be significantly different among gender.

**Table 1:** The independent sample *T*-test of the gender and age among the model variables.

Variables	*N*	CPA		RS		RV		RA		CR	
		*M* ± *SD*	*t/F*	*M* ± *SD*	*t/F*	*M* ± *SD*	*t/F*	*M* ± *SD*	*t/F*	*M* ± *SD*	*t/F*
*Age*			0.17***		0.03		0.15***		−0.08		−0.14***
12–14 years	901	0.80 ± 0.68		7.22 ± 4.08		1.36 ± 0.52		0.75 ± 0.82		3.39 ± 0.97	
15–17 years	967	0.68 ± 0.56		7.19 ± 3.77		1.23 ± 0.42		0.80 ± 0.65		3.61 ± 0.77	
*Gender*			−0.31**		−0.28***		−0.31		−0.06		−0.10
Male	923	0.69 ± 0.59		6.51 ± 3.49		1.28 ± 0.47		0.77 ± 0.78		3.48 ± 0.93	
Female	945	0.78 ± 0.66		7.89 ± 4.19		1.30 ± 0.48		0.78 ± 0.69		3.52 ± 0.83	

**p < 0.01, ***p < 0.001. CPA, Childhood psychological abuse; RS, Rejection sensitivity; RV, Relational victimization; RA, Relational aggression; CR, Cognitive reappraisal.

Table 2 showed the correlation analysis results among childhood psychological abuse, rejection sensitivity, relational victimization, relational aggression, and cognitive reappraisal. Childhood psychological abuse was positively correlated with rejection sensitivity (*r* = 0.34, *p* < 0.01), relational victimization (*r* = 0.42, *p* < 0.01), and relational aggression (*r* = 0.22, *p* < 0.01). Rejection sensitivity was positively correlated with relational victimization (*r* = 0.33, *p* < 0.01) and relational aggression (*r* = 0.27, *p* < 0.01). Relational victimization was positively correlated with relational aggression (*r* = 0.22, *p* < 0.01). Negative correlations were observed between cognitive reappraisal and childhood psychological abuse (*r* = −0.12, *p* < 0.01), rejection sensitivity (*r* = −0.16, *p* < 0.01), relational victimization (*r* = −0.13, *p* < 0.01), and relational aggression (*r* = −0.19, *p* < 0.01), respectively. Both childhood psychological abuse (*r* = 0.08, *p* < 0.01) and rejection sensitivity (*r* = 0.18, *p* < 0.01) were positively associated with gender. Age was adversely correlated with childhood psychological abuse (*r* = −0.05, *p* < 0.05) and relational victimization (*r* = −0.12, *p* < 0.01). Both relational aggression (*r* = 0.07, *p* < 0.01) and cognitive reappraisal (*r* = 0.13, *p* < 0.01) were positively correlated with age.

**Table 2:** Descriptive statistics of model variables and correlations among model variables.

Variables	*M ± SD*	CPA	RS	RV	RA	CR
CPA	0.74 ± 0.62	−				
RS	7.21 ± 3.92	0.34**	−			
RV	1.29 ± 0.47	0.42**	0.33**	−		
RA	0.78 ± 0.74	0.22**	0.27**	0.22**	−	
CR	3.50 ± 0.88	−0.12**	−0.16**	−0.13**	−0.19**	−
Gender	0.51 ± 0.50	0.08**	0.18**	0.02	0.01	0.02
Age	14.31 ± 1.60	−0.05*	0.00	−0.12**	0.07**	0.13**

*p < 0.05, **p < 0.01. Gender: 0 = male, 1 = female. CPA, Childhood psychological abuse; RS, Rejection sensitivity; RV, Relational victimization; RA, Relational aggression; CR, Cognitive reappraisal.

### Testing the mediating effects of rejection sensitivity and victimization on childhood psychological abuse and relational aggression

The Process plug-in (Model 6) in SPSS compiled by Hayes (2017) was used to test the chain mediating effect of rejection sensitivity and relational victimization on childhood psychological abuse and relational aggression with gender and age serving as control variables. As shown in Table 3, the direct effect of childhood psychological abuse on relational aggression was significant (*β* = 0.23, *p* < 0.001), and remained significant (*β* = 0.11, *p* < 0.001) after the inclusion of two mediating variables, rejection sensitivity and relational victimization. Childhood psychological abuse was significant positively related to rejection sensitivity (*β* = 0.33, *p* < 0.001); rejection sensitivity was significantly positive related to relational victimization (*β* = 0.22, *p* < 0.001), which in turn was positively related to relational aggression (*β* = 0.12, *p* < 0.001). The findings suggest that childhood psychological abuse was significantly linked with relational aggression *via* the chain mediated effect of rejection sensitivity and relational victimization. Meanwhile, childhood psychological abuse was significantly positive related to relational victimization (*β* = 0.34, *p* < 0.001). Rejection sensitivity (*β* = 0.20, *p* < 0.001) and relational victimization (*β* = 0.12, *p* < 0.001) were significantly related to relational aggression. The findings suggest that childhood psychological abuse was significantly linked with relational aggression *via* the separate mediated effect of rejection sensitivity and relational victimization.

**Table 3:** Regression analysis of variables in the model.

Regression equation		Overall fitting index			SORC			
Outcome	Predictor	*R*	*R* ^2^	*F*	*β*	95% CI LI	95% CI UI	*t*
RA	CPA	0.24	0.06	37.21***	0.23	0.18	0.27	10.10***
	Gender				−0.01	−0.06	0.03	−0.51
	Age				0.08	0.04	0.13	3.59***
RS	CPA	0.37	0.14	99.28***	0.33	0.29	0.37	15.19***
	Gender				0.15	0.11	0.19	6.98***
	Age				0.02	−0.03	0.06	0.68
RV	RS	0.47	0.22	133.39***	0.22	0.18	0.26	9.91***
	CPA				0.34	0.30	0.38	15.61***
	Gender				−0.04	−0.08	−0.01	−2.02*
	Age				−0.10	−0.14	−0.06	−4.85***
RA	RS	0.33	0.11	46.61***	0.20	0.16	0.25	8.36***
	RV				0.12	0.07	0.16	4.65***
	CPA				0.11	0.07	0.16	4.64***
	Gender				−0.04	−0.08	0.02	−1.84
	Age				0.09	0.05	0.13	4.04***

*p < 0.05, **p < 0.01, ***p < 0.001. Before substituting into the equation, all variables in the model were standardized. 95% CI LI, 95% CI lower limits; 95% CI UI, 95% CI upper limits; SORC, significance of regression coefficient; CPA, Childhood psychological abuse; RS, Rejection sensitivity; RV, Relational victimization; RA, Relational aggression; CR, Cognitive reappraisal.

**Figure 2:**
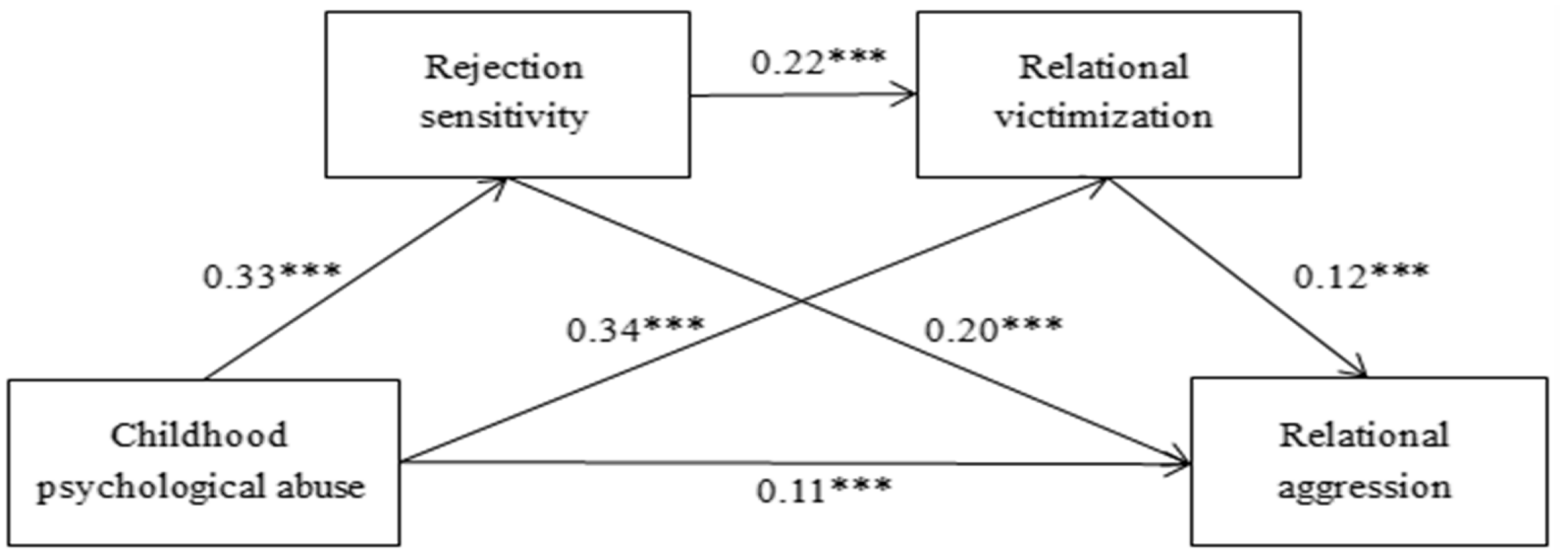
The chain mediating effect of rejection sensitivity and relational victimization, ***p <0.001.

The results of the additional mediating effect test (shown in Table 4; Figure 2) stated that childhood psychological abuse could influence relational aggression *via* the separate mediating effects of rejection sensitivity and relational victimization, as well as *via* the chain mediating effects of rejection sensitivity and relational victimization. In particular, the mediating effect is generated through three pathways: indirect pathway 1, childhood psychological abuse → rejection sensitivity →relational aggression; indirect pathway 2, childhood psychological abuse →relational victimization → relational aggression; indirect pathway 3, childhood psychological abuse →rejection sensitivity →relational victimization →relational aggression. The indirect effect of the three indirect paths does not contain 0 in the 95% confidence interval of Bootstrap, reflecting that the indirect effect of the three indirect paths has achieved a significant level, and the mediation effect accounts for 29, 17 and 4% of the aggregate effect, respectively.

**Table 4:** The chain mediating effect of rejection sensitivity and relational victimization.

Effect	Pathway	Effect value	95% CI LI	95% CI UI	RME
Direct effect	CPA → RA	0.115	0.184	0.273	50%
Mediation effect	CPA → RS → RA	0.066	0.049	0.088	29%
	CPA → RV → RA	0.039	0.020	0.060	17%
	CPA → RS → RV → RA	0.008	0.004	0.014	4%
Total effect		0.228	−	−	100%

95% CI LI, 95% CI lower limits; 95% CI UI, 95% CI upper limits; RME, Relative mediating effect; CPA, Childhood psychological abuse; RS, Rejection sensitivity; RV, Relational victimization; RA, Relational aggression; CR, Cognitive reappraisal.

### Test of moderating effect of cognitive reappraisal

**Figure 3:**
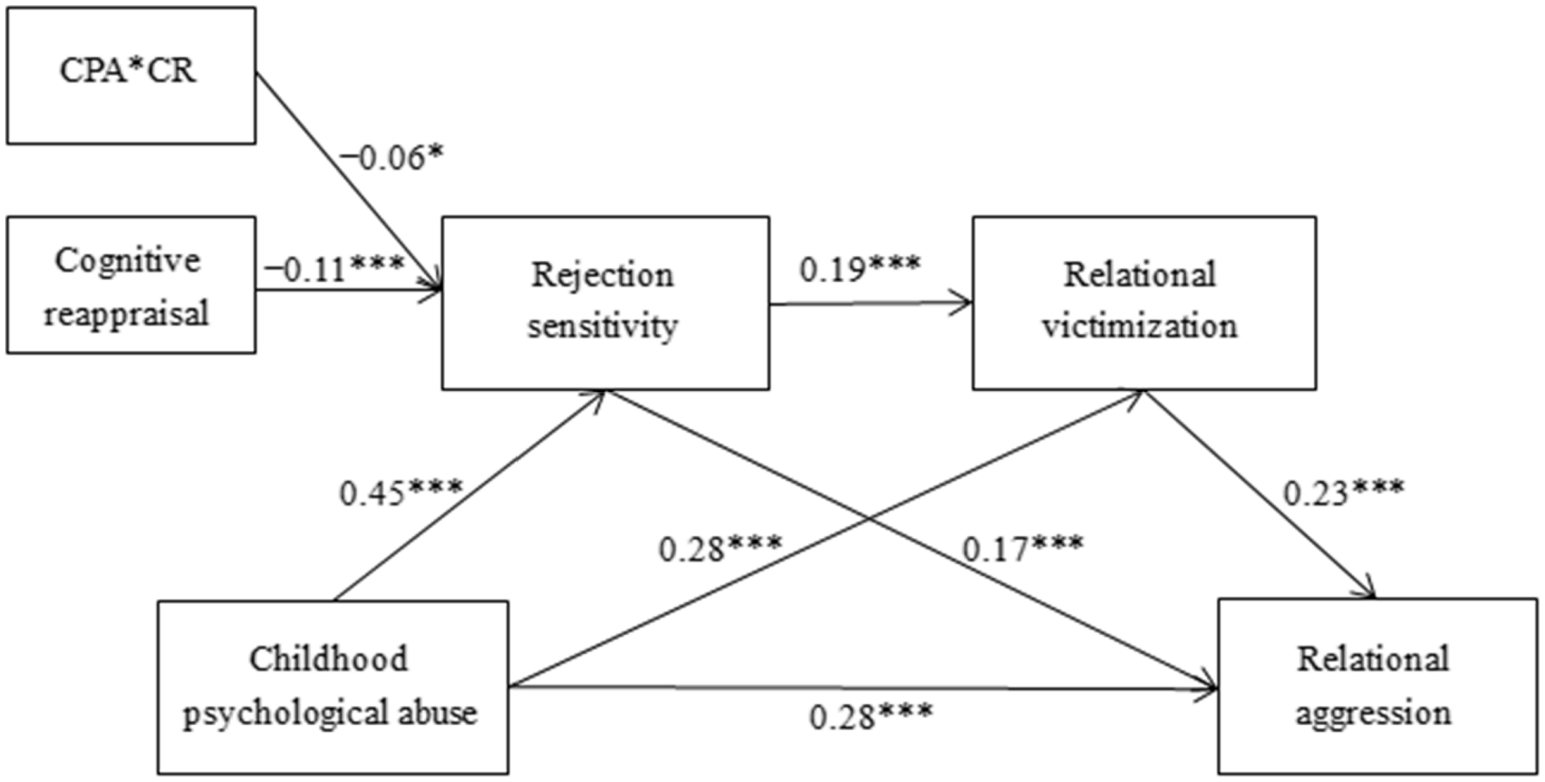
The moderated mediation model, gender and age are not represented in the model for concise purposes, *p < 0.05, ***p < 0.001.

According to the suggestions of Wen and Ye (2014), the moderated chain mediation model was constructed using Mplus7.4 with childhood psychological abuse as the independent variable, relational aggression as the dependent variable, rejection sensitivity and relational victimization as the mediating variables, cognitive reappraisal as the moderating variable, and gender and age as the control variables. The results showed that the model fitted well: *χ*^2^ = 60.272, *df* = 11, CFI = 0.961, TLI = 0.909, RMSEA = 0.049, SRMR = 0.022, indicating that the model was acceptable. Controlling for gender and age, the interaction effect between cognitive reappraisal and childhood psychological abuse on rejection sensitivity was significant (*β* = −0.06, *p* < 0.05, 95% CI = [−0.068, −0.047]), highlighting that cognitive reappraisal may moderate the effect of childhood psychological abuse on rejection sensitivity (shown in Figure 3). Considering that the correlation between cognitive reappraisal and other variables may lead to deviation of the results of the study, the multicollinearity test was conducted. The results showed that the VIF values of childhood psychological abuse, rejection sensitivity, relational victimization, cognitive reappraisal and the interaction terms of childhood psychological abuse and cognitive reappraisal were 1.258, 1.202, 1.276, 1.037 and 1.012, respectively, all of which were less than the critical value 5 (Wen et al., 2018), and there was no multicollinearity problem.

**Figure 4:**
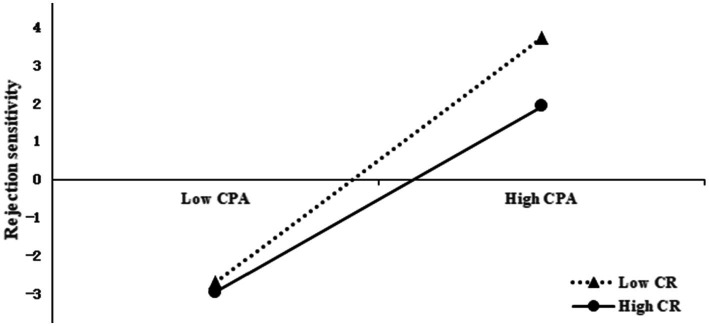
The moderating effect of cognitive reappraisal on childhood psychological abuse and rejection sensitivity.

To further reveal the moderating effect of cognitive reappraisal in the chain mediation path, this study divided cognitive reappraisal into high group (*M* + 1*SD*) and low group (*M* − 1*SD*), and calculated the effect value of childhood psychological abuse on rejection sensitivity under different levels of cognitive reappraisal, so as to draw a simple slope analysis figure (Figure 4). Simple slope tests showed that childhood psychological abuse was significantly linked with adolescents’ rejection sensitivity in high and low-level cognitive reappraisal, but the relationship between childhood psychological abuse and rejection sensitivity in adolescents with low cognitive reappraisal level (*b*_simple_ = 0.01, *p* < 0.001, 95% CI = [0.006, 0.015]) was significantly stronger than that in adolescents with high cognitive reappraisal level (*b*_simple_ = 0.006, *p* < 0.001, 95% CI = [0.003, 0.01]). The results indicate that the relationship between childhood psychological abuse and rejection sensitivity was moderated by cognitive reappraisal.

### Alternate model analyses

Based on the general aggression model and developmental systems theory, this study hypothesizes that childhood psychological abuse was significantly linked with relational aggression through the chain mediating effects of rejection sensitivity and relational victimization (CPA → RS → RV → RA). For greater confidence in the proposed direction of associations between the childhood psychological abuse and relational aggression, three alternative models were tested to control the timeframe that was being asked in each questionnaire. The results (shown in Table 5) indicate that hypothesizes model (*χ*^2^_(2)_ = 7.664, CFI = 0.994, TLI = 0.972, RMSEA = 0.039 [0.013, 0.07], SRMR = 0.016) display good model fit and provide stronger evidence for the chain mediating pathways proposed.

**Table 5:** The model fit comparisons between hypothetical theoretical model and alternative model.

Model	*χ* ^2^	*df*	CFI	TLI	RMSEA	SRMR
CPA → RS → RV → RA(Hypothesizes Model)	7.664	2	0.994	0.972	0.039	0.016
CPA → RS → RA → RV(Alternate Model 1)	52.222	2	0.945	0.754	0.116	0.04
CPA → RA → RV → RS(Alternate Model 2)	56.02	2	0.941	0.735	0.12	0.039
CPA → RV → RS → RA(Alternate Model 3)	55.816	2	0.941	0.736	0.12	0.04

CPA, Childhood psychological abuse; RS, Rejection sensitivity; RV, Relational victimization; RA, Relational aggression; CR, Cognitive reappraisal.

Some empirical studies have found that relational aggression is a predictor of and relational victimization (Schmidt and Jankowski, 2014), and there was an interactive relationship between relational victimization and rejection sensitivity (Nicole et al., 2018). Therefore, we propose three alternative models: childhood psychological abuse (CPA) → rejection sensitivity (RS) → relational aggression (RA) → relational victimization (RV); childhood psychological abuse (CPA) → relational aggression (RA) → relational victimization (RV) → rejection sensitivity (RS); and childhood psychological abuse (CPA) → relational victimization (RV) → rejection sensitivity (RS) → relational aggression (RA). The results (shown in Table 5) indicate that alternative model 1 (*χ*^2^_(2)_ = 52.222, CFI = 0.945, TLI = 0.754, RMSEA = 0.116 [0.09, 0.144], SRMR = 0.04), alternative model 2 (*χ*^2^_(2)_ = 56.02, CFI = 0.941, TLI = 0.735, RMSEA = 0.12 [0.094, 0.148], SRMR = 0.039), and alternative model 3 (*χ*^2^_(2)_ = 55.816, CFI = 0.941, TLI = 0.972, RMSEA = 0.12 [0.094, 0.148], SRMR = 0.04) display not well model fit. The above results is well-supported the hypothetical model, but potential reciprocal relationships among childhood psychological abuse, rejection sensitivity, relational victimization and relational aggression cannot be ruled out.

## Discussion

The study found that childhood psychological abuse was significantly positive linked with relational aggression among adolescents, which means that adolescents who have suffered more childhood psychological abuse will have a higher level of relational aggression. The results accord with the general aggression model and spillover hypothesis, adolescents tend to apply the psychological abuse they learn from their parents in childhood to interpersonal situations, thus causing aggression. At the same time, in order to understand the internal mechanism of childhood psychological abuse and relational aggression among adolescents. This study proposed a moderated chain mediation model based on general aggression model, attachment theory, frustration-aggression theory, interpersonal risk model, developmental systems theory, and emotion regulation process model to investigate the internal mechanism.

### The chain mediating effect of rejection sensitivity and relational victimization

The findings indicate that childhood psychological abuse was significantly positive linked with adolescents’ relational aggression through the separate mediating effects of rejection sensitivity and relational victimization, as well as through the chain mediating effects of rejection sensitivity and relational victimization. This illustrates that rejection sensitivity and relational victimization are the key factors for the development of negative coping styles of adolescents who have experienced childhood psychological abuse. Preceding empirical research has demonstrated that rejection sensitivity mediates the relationship between childhood abuse and aggression (Liu et al., 2018). Individuals residing in unhealthy familial contexts are more susceptible to peer victimization in peer communication (Shields and Cicchetti, 2001; Hong and Espelage, 2012), hence increasing the frequency of aggressive behavior (Sullivan et al., 2006; Light et al., 2013). This further indicates that there are important influencing variables between childhood psychological abuse and relational aggression. Specifically, the intrinsic traits (rejection sensitivity) and peer relationship factors (relational victimization) of individuals who endured childhood psychological abuse are the significant determining variables that contribute to relational aggression in adolescents.

According to attachment theory and frustration-aggression theory, childhood psychological abuse can impair adolescents’ rejection sensitivity, which in turn influences their relational aggression behavior. On the one hand, adolescents who have withstood long-term psychological abuse (such as intimidation and rejection) find it difficult to establish a sense of security in the interpersonal environment and become especially sensitive to rejection cues in the interpersonal environment (Natarajan et al., 2011). On the other hand, individuals who are sensitive to rejection cues experience interpersonal frustration for an extended period of time, thereby triggering aggression (Bondü and Krahé, 2015). This is also consistent with preceding empirical research that adolescent rejection sensitivity is influenced by early traumatic experience, attachment pattern, and parenting style (Zheng et al., 2020), and that rejection sensitivity can lead to a variety of problematic behaviors (Romero-Canyas et al., 2010). Therefore, childhood psychological abuse can escalate relational aggression in adolescence by heightening their rejection sensitivity. According to attachment theory and the interpersonal risk model, however, adolescents who experienced childhood psychological abuse have difficulty in establishing a secure attachment mode and are more likely to express negative emotions and hostile reactions in interpersonal communication, thereby suffering from peer relationship victimization (Zhu et al., 2019). Individuals who experience relational victimization are inclined to respond with relationship aggression in order to defend themselves from the harm in the interpersonal environment. Additionally, prior empirical research has demonstrated that relational victimization can predict relational aggression (Ji et al., 2012). Hence, as a subtype of peer victimization, relational victimization is another crucial factor driving adolescent relational aggression following childhood psychological abuse.

The study also discovered that rejection sensitivity and relational victimization mediated the association between childhood psychological abuse and relational aggression, and it investigated the internal mechanism between childhood psychological abuse and relational aggression. Individual’s internal personality inclination and companion factors are two significant elements that influence the development of adolescents. According to developmental systems theory, individuals who endure a poor family environment characterized by childhood psychological abuse are more sensitive to cues of rejection and hostility in the interpersonal context because they frequently receive negative responses from their parents (Brendgen et al., 2002). This overreaction to interpersonal cues renders individuals more susceptible to victimization by peers (Rudolph et al., 2008). Individuals who are victimized by their peers in turn become the subject of relational aggression (Sullivan et al., 2006).

### The moderating effect of cognitive reappraisal

The moderating role of cognitive reappraisal in the chain-mediated pathway of rejection sensitivity and relational victimization was also investigated. The results indicate that cognitive reappraisal can impact the association between childhood psychological abuse and rejection sensitivity. Cognitive reappraisal mitigated the negative effects of childhood psychological abuse on rejection sensitivity and further attenuates the indirect effects of childhood psychological abuse and relational aggression. This result again supports the emotional regulation process model and gives strong evidence for demonstrating that cognitive reappraisal acts as a buffer in the influence of negative life events on individuals. When presented with a similar home setting of psychological abuse, individuals who are adept at employing cognitive reappraisal strategies are able to see the event positively and lessen the negative emotional experience and negative cognition brought on by the harsh environment (Gross and John, 2003). Secondly, individuals with high frequency of cognitive reappraisal can continuously strengthen their positive cognition and emotion to environmental cues, and reduce their sensitivity to interpersonal environment rejection cues, thereby lessening the negative impact of childhood psychological abuse on adolescents’ rejection sensitivity.

## Conclusion

Childhood psychological abuse was significant positively related to adolescents’ relational aggression. The severity of their childhood psychological abuse was proportional to their level of relational aggression. Childhood psychological abuse was significantly linked with adolescents’ relational aggression through the separate mediating effects of rejection sensitivity and relational victimization, as well as through the chain mediating effects of rejection sensitivity and relational victimization. Cognitive reappraisal moderated the chain mediating effect of childhood psychological abuse on adolescent relational aggression *via* rejection sensitivity and relational victimization.

### Implications for theory and practice

This study investigated the internal mechanism of childhood psychological abuse on adolescent relational aggression by constructing a moderated chain mediation model, revealing the separate mediating roles of rejection sensitivity and relational victimization, as well as the chain mediating roles of both. In light of this, we investigate further the moderating effect of cognitive reappraisal on the link between childhood psychological abuse and rejection sensitivity, which provides useful insight for mitigating the harmful effects of childhood psychological abuse on adolescent development.

First and foremost, we should be mindful of our parenting style and strive to avoid shaping children’s behavior patterns through intimidation, denigration, and neglect. Secondly, parents and educators should timely pay attention to the personality tendency of adolescents’ rejection sensitivity and intervene with rational emotive therapy. The findings suggest that cognitive reappraisal can mitigate the negative effects of childhood psychological abuse on rejection sensitivity. Therefore, educators can train students to use positive emotion regulation strategies in the form of psychology classes, class meetings and group tutorials, enhance students’ positive cognition, learn to identify and adjust their unreasonable cognition, so as to correct students’ attribution bias, help students understand rejection and how to properly deal with the negative emotions caused by rejection. Finally, it is necessary to find out the relational victimization phenomenon in time and reduce the frequency of this phenomenon. It is difficult to identify relational victims because of the invisible nature of relational victimization. Parents can build connections and strengthen communication with adolescents so that the children can gain support and strength in the family to avoid the situation of isolation after the relational victimization. Psychology teachers can develop some experiential courses or group counseling programs related to interpersonal relationship and campus violence, identify victims from observation, and provide timely help and support to students who have been victimized by relationships, so as to reduce the possibility of their transformation into relational aggressors.

### Limitations and future study

First of all, the variables in this study were measured by subjective assessment of adolescents, which may hold some deviation. Peer nomination, parent/teacher report, and teenage self-evaluation can be merged in the future to provide multifaceted evidence for the data. Second, teenage relational aggression is highly variable, making it challenging to determine the causal relationship between variables using a cross-sectional design alone. In the future, longitudinal studies should be considered to further investigate the dynamic development of relational aggression and its causal link with other variables. Thirdly, rejection sensitivity is not only influenced by external factors such as childhood psychological abuse, but also by individual internal factors such as self-esteem. Future studies should comprehensively consider the influence of internal and external factors on rejection sensitivity. Finally, this study only addressed the moderating effect of cognitive reappraisal; for a more comprehensive discussion, additional forms of emotion regulation strategies can be included in future research.

## Data availability statement

The raw data supporting the conclusions of this article will be made available by the authors, without undue reservation.

## Ethics statement

The studies involving human participants were reviewed and approved by the Ethics in Human Research Committee of School of Education, Guangzhou University. Written informed consent to participate in this study was provided by the participants’ legal guardian/next of kin.

## Author contributions

TL, YH, and YM were involved in the conceptualization of the research project and supervised the data collection. TL and YH were responsible for data analysis and report writing. MJ, SM, and YM provided feedback on the manuscript. All authors contributed to the article and approved the submitted version.

## Funding

This study was supported by Guangzhou Education Science Planning Project: Research on Students’ Emotional Intelligence based on the Perspective of Intergenerational Transmission, grant number: 202113497.

## Conflict of interest

The authors declare that the research was conducted in the absence of any commercial or financial relationships that could be construed as a potential conflict of interest.

## Publisher’s note

All claims expressed in this article are solely those of the authors and do not necessarily represent those of their affiliated organizations, or those of the publisher, the editors and the reviewers. Any product that may be evaluated in this article, or claim that may be made by its manufacturer, is not guaranteed or endorsed by the publisher.

## Footnotes

^1^ http://www.afhayes.com
